# A Projectile Concussive Impact Model Produces Neuroinflammation in Both Mild and Moderate-Severe Traumatic Brain Injury

**DOI:** 10.3390/brainsci13040623

**Published:** 2023-04-06

**Authors:** Lindsay T. Michalovicz, Kimberly A. Kelly, Travis J. A. Craddock, James P. O’Callaghan

**Affiliations:** 1 Health Effects Laboratory Division, National Institute for Occupational Safety and Health, Centers for Disease Control and Prevention, Morgantown, WV 26508, USA; 2 Institute for Neuro-Immune Medicine, Nova Southeastern University, Fort Lauderdale, FL 33314, USA; 3 Department of Clinical Immunology, College of Osteopathic Medicine, Nova Southeastern University, Fort Lauderdale, FL 33314, USA; 4 Department of Psychology & Neuroscience, College of Psychology, Nova Southeastern University, Fort Lauderdale, FL 33314, USA; 5 Department of Computer Science, College of Engineering and Computing, Nova Southeastern University, Fort Lauderdale, FL 33314, USA

**Keywords:** traumatic brain injury, inflammation, projectile concussive impact, neural damage, logic modeling, neuroimmune

## Abstract

Traumatic brain injury (TBI) is a major cause of death and disability and is experienced by nearly 3 million people annually as a result of falls, vehicular accidents, or from being struck by or against an object. While TBIs can range in severity, the majority of injuries are considered to be mild. However, TBI of any severity has the potential to have long-lasting neurological effects, including headaches, cognitive/memory impairments, mood dysfunction, and fatigue as a result of neural damage and neuroinflammation. Here, we modified a projectile concussive impact (PCI) model of TBI to deliver a closed-head impact with variable severity dependent on the material of the ball-bearing projectile. Adult male Sprague Dawley rats were evaluated for neurobehavioral, neuroinflammatory, and neural damage endpoints both acutely and longer-term (up to 72 h) post-TBI following impact with either an aluminum or stainless-steel projectile. Animals that received TBI using the stainless-steel projectile exhibited outcomes strongly correlated to moderate-severe TBI, such as prolonged unconsciousness, impaired neurobehavior, increased risk for hematoma and death, as well as significant neuronal degeneration and neuroinflammation throughout the cortex, hippocampus, thalamus, and cerebellum. In contrast, rats that received TBI with the aluminum projectile exhibited characteristics more congruous with mild TBI, such as a trend for longer periods of unconsciousness in the absence of neurobehavioral deficits, a lack of neurodegeneration, and mild neuroinflammation. Moreover, alignment of cytokine mRNA expression from the cortex of these rats with a computational model of neuron–glia interaction found that the moderate-severe TBI produced by the stainless-steel projectile strongly associated with the neuroinflammatory state, while the mild TBI existed in a state between normal and inflammatory neuron–glia interactions. Thus, these modified PCI protocols are capable of producing TBIs that model the clinical and experimental manifestations associated with both moderate-severe and mild TBI producing relevant models for the evaluation of the potential underlying roles of neuroinflammation and other chronic pathophysiology in the long-term outcomes associated with TBI.

## 1. Introduction

Approximately 1.4–2.5 million people in the United States experience a traumatic brain injury (TBI) each year, and in 2020, over 60,000 deaths were attributed to TBI [1,2,3,4,5]. TBIs are typically separated into two major categories: moderate-severe (sTBI) and mild (mTBI)/concussion. While more severe TBI can result in hospitalization or death, the majority (>70%) of TBIs are mild [3,6]. sTBI usually results from impacts that deliver higher forces upon the head and/or the brain compared to mTBI, including penetrating injuries, and produces more severe effects, including coma, amnesia, cognitive, motor, and sensory issues, depression, anxiety, other mood disorders, and death. In contrast, mTBI may be difficult to detect due to the lack of a clear clinical definition and the presence of mild and more transient symptoms [7]. Despite these differences, the incidence of TBI of any severity is associated with an increased risk for long-term disability and mortality [2,8,9]. However, the underlying cause of these long-term consequences largely remains unclear.

Many studies have shown that neuroinflammation occurs following TBI as a secondary response to damaging the brain [2,10,11,12]. In sTBI, this can become a cyclical process where damage to the brain activates the brain’s resident immune response and allows for the infiltration of peripheral immune cells, which may facilitate further damage to the tissues and expand the size of the injury. However, studies have shown that even the axonal shearing and stretching that can result from mTBI may produce neuroinflammation [8]. In this case, the response is likely more restricted to the brain’s immune cells (e.g., astrocytes and microglia), which can also be directly damaged as a result of TBI [12]. While the significant damage associated with sTBI logically produces an increased risk for long-term neurological dysfunction, mTBI is also capable of producing long-term health effects, which are emphasized by the association of repeated mTBIs with long-term diseases such as Alzheimer’s Disease (AD), Parkinson’s Disease (PD), neuropsychiatric disorders, cognitive dysfunction, and chronic traumatic encephalopathy (CTE) [8,9,13,14,15,16,17,18,19,20,21,22]. Moreover, several studies that investigated the impacts of repeated mTBIs have indicated increased neuroinflammatory responses concurrent with neurocognitive changes [23,24,25,26,27,28,29]. Thus, it is crucial to understand the role that neuroinflammation may play in TBI and how it may impact long-term health outcomes and future neurological diseases.

Many experimental models of TBI exist, including weight drop, fluid-percussion, blast, and controlled cortical impact; however, a major caveat of these common methods is that they require a craniotomy in order to be performed [30]. This presents a problem when focusing on neuroinflammation as an outcome of TBI, as surgery and the neuroactive anesthetics needed to perform them can have secondary impacts on the brain’s immune response beyond the TBI itself. A novel model of TBI that circumvents the need for craniotomy is the projectile concussive impact (PCI) model developed at the Walter Reed Army Institute of Research (WRAIR), which produces a closed-head impact [23,25,28,31,32]. To produce these effects without compromising the integrity of the skull, a helmet is used to protect the head and disperse the transferred forces of the ball bearing [23]. However, there are many scenarios in which a TBI may occur when a helmet is not present, and the use of a helmet in this model precludes its use to test prospective helmet materials for improved safety in both recreational- and occupational-use helmets.

Thus, in this current study, our goal was to modify the WRAIR PCI model of TBI to develop a model of TBI, specifically mTBI, that would not require a helmet to protect against penetrating damage to the skull while still producing sequelae relevant to incidents of mTBI. By varying the material of the ball bearing from stainless steel to aluminum, we were able to produce a model of mTBI that results in mild incidences of unconsciousness and activation of astrocytes and microglia compared to the sTBI produced by the stainless-steel ball bearing. Moreover, by aligning the outcomes from the mTBI and sTBI models with an existing logic model of neuroimmune activation [33], we found that sTBI strongly associates with a neuroinflammatory state, whereas our mTBI model falls in between the neuroinflammatory and healthy model states. These findings suggest that despite the milder and more transient effects associated with mTBI, the condition is capable of producing neuroinflammation, which could be the seed of future neurological dysfunction, particularly when considering the potential cumulative effects in individuals experiencing multiple mTBIs.

## 2. Materials and Methods

### 2.1. Animals

All procedures were performed within protocols approved by the Centers for Disease Control and Prevention-Morgantown Institutional Animal Care and Use Committee in an AAALAC International accredited facility. A total of 30 4-month-old adult male Sprague Dawley rats (*N* = 30) weighing approximately 250–300 g were purchased from Hilltop Lab Animals, Inc. (Scottdale, PA, USA). Upon arrival, rats were group housed (2 rats/cage) in a temperature (21 ± 1 °C) and humidity (50 ± 15%) controlled room maintained under filtered positive-pressure ventilation on a 12 h light/dark cycle (lights on 0600 ET) in the CDC-Morgantown Animal Facility and allowed to acclimate for 1 week prior to the experimental TBI procedure. Rats were given ad libitum access to food (Teklad 2918 Global 18% rat chow; Envigo, Madison, WI, USA) and water and received daily health checks from animal husbandry personnel. Rats were arbitrarily divided into 3 groups (*N* = 10/group): sham, mild TBI, and severe TBI.

### 2.2. PCI Model of TBI

**Figure 1:**
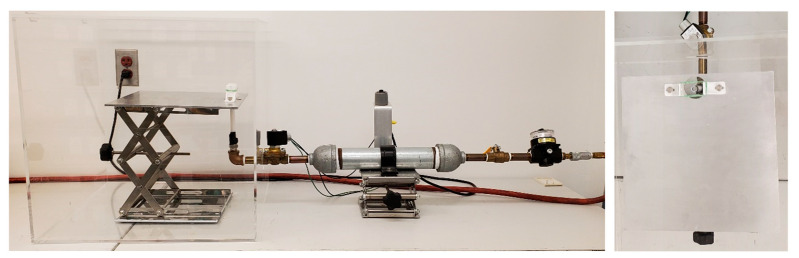
Projectile Concussive Impact device.

The PCI model of TBI was originally developed at WRAIR [23,31]. For this study, we performed a modified PCI exposure procedure using a PCI device constructed similarly to the device described in Leung et al., 2014 (Figure 1) without the use of a helmet. Prior to PCI, rats were anesthetized using an E-Z Anesthesia isoflurane vaporizer with a Sure-Seal shoebox induction chamber (E-Z Systems, Palmer, PA, USA) with 4–5% isoflurane for induction until unconscious and then the concentration was reduced to 2% for a total of approximately 4 min. Following anesthesia, the rat was removed from the induction chamber and immediately placed supine on the PCI device platform with the top of the head placed above the circular opening in the platform and gently secured in place with a rubber band to prevent head rotation/movement prior to the TBI impact. The barrel of the PCI device was aimed approximately equidistant between the eyes and ears (front to back) and centered (left to right). A ball bearing was propelled at the top of the head using 20 PSI of air pressure; mild and severe TBIs were instigated with either an aluminum (mass approximately 0.34 g) or stainless-steel (mass approximately 3.47 g) ball bearing, respectively. Sham animals underwent all of the same procedures as mild and severe TBI except that no ball bearing was loaded into the PCI device. Following impact, the rats were immediately removed from the PCI device enclosure and returned to their home cage for recovery. The “time to recovery” was recorded for each animal as the elapsed time from removal from the anesthesia induction chamber to regaining consciousness in their home cage. Pressure film (Medium (MS) Fujifilm Prescale Tactile Pressure Indicating Sensor Film, Fujifilm, Tokyo, Japan) was affixed to the PCI platform by taping one edge over the opening to estimate impact forces. Pressure films were analyzed using the Topaq Pressure Analysis System (Sensor Products Inc., Madison, NJ, USA). The aluminum and stainless-steel ball bearings were estimated to produce impact forces of 18.84 ± 3.69 and 181.12 ± 18.51 N, respectively. The impact force measured for the stainless-steel ball bearing is similar to the impact forces reported for those transferred to the inside of the helmet by Leung et al. [23]. 

### 2.3. Neurobehavioral Severity Scoring

All rats were subjected to the Revised Neurobehavioral Severity Scale (NSS-R), as described previously [34], at baseline the day prior to TBI and 1, 6, 24, 48, and 72 h following TBI, depending on the tissue collection time point (i.e., 6 or 72 h post-TBI). Briefly, performance was scored using a Likert scale of 0 to 2 on a battery of 10 tests, including general balance, landing, tail raise, drag, righting reflex, ear reflex, eye reflex, sound, reflex, tail reflex, and paw flexion reflex. Upon completion of initial baseline testing, it was determined that the rats included in this study did not produce a “normal” response to the ear reflex, tail reflex, or paw flexion reflex tests; these tests were omitted from cumulative scoring. The individual scores on each remaining test were summed to produce a cumulative neurobehavioral score at each time point tested. All rats behaved normally (0 score) at baseline. 

### 2.4. Tissue Collection

Each of the three TBI exposure groups (Sham, mTBI, and severe TBI) were arbitrarily separated into two subgroups (*N* = 5/group) for the collection of tissue at 2 time points: 6 and 72 h post-TBI. Rats (*N* = 5/group) were euthanized at 6 h post-TBI by decapitation to evaluate cytokine mRNA expression by qPCR. Briefly, whole brains were removed from the skull and dissected free-hand on a thermoelectric cold plate, as previously described [35], to isolate cortex, hippocampus, striatum, and cerebellum tissues. For this study, the cortex was subdivided into 3 pieces by hemisphere: frontal, medial, and posterior. Isolated brain tissue samples were immediately placed on dry ice and stored at −80 °C. The remaining rats (*N* = 5/group) were euthanized at 72 h post-TBI by terminal formalin perfusion. Briefly, rats were deeply anesthetized with pentobarbital-based euthanasia solution (Fatal Plus, Vortech Pharmaceuticals, Dearborn, MI, USA) until unresponsive and then transcardially perfused with 0.1 M Phosphate Buffered Saline (PBS) (FD Neurotechnologies, Inc., Colombia, MD, USA) followed by 4% paraformaldehyde (PFA) in 0.1 M PBS (FD Neurotechnologies, Inc., Colombia, MD, USA). Following perfusion, the rat brains were carefully removed from the skull and post-fixed in 4% PFA at 4 °C for a minimum of 6 h.

### 2.5. Immunofluorescence

Following post-fixation with PFA, brains were cryopreserved in 20% sucrose in 0.1 M PBS (FD Neurotechnologies, Inc., Colombia, MD, USA) and shipped to FD Neurotechnologies, Inc. for processing. Briefly, 30 μm coronal sections were mounted on slides and stained with Fluro-Jade B (FD Neurotechnologies, Inc., Colombia, MD, USA) or co-stained with GFAP (1:800; BD Biosciences, San Jose, CA, USA; Cat# 556330) and Iba1 (1:1000; Wako, Osaka, Japan; Cat# 019 19741). Sections were visualized using an Olympus BX 63 microscope (Center Valley, PA, USA), and images were captured using Cell Sens Dimension software with an Olympus DP 73 digital camera attached to the microscope. Images were captured at 10× magnification unless specified otherwise.

### 2.6. qPCR

Frozen brain tissues were thawed and homogenized in Trizol Reagent (Thermo Fisher Scientific, Waltham, MA, USA) to isolate total RNA for reverse transcription to cDNA and subsequent qRT-PCR analysis, as previously described [36,37]. Briefly, a subsample of tissues collected from the cortical areas, hippocampus, and cerebellum, as well as one hemisphere of the striatum, were reverse transcribed using Superscript III reactions (Thermo Fisher Scientific, Waltham, MA, USA). qRT-PCR of the housekeeping gene glyceraldehyde-3-phosphate dehydrogenase (GAPDH), and target genes: tumor necrosis factor-alpha (TNFα), interleukin 6 (IL-6), C–C chemokine ligand 2 (CCL2), interleukin 1beta (IL-1β), leukemia inhibitor factor (LIF), oncostatin M (OSM), and glial fibrillary acidic protein (GFAP), was performed using an Applied Biosystems 7500 real-time PCR system (Thermo Fisher Scientific, Waltham, MA, USA) in combination with TaqMan^®^ chemistry. The mRNA expression fold changes for TNFα, IL-6, CCL2, IL-1β, LIF, OSM, and GFAP were determined using the ΔΔCt method with normalization to GAPDH. All values are expressed as relative fold change normalized to Sham controls.

### 2.7. Discrete Logic Modeling of Neuron-Glia Interaction

**Figure 2:**
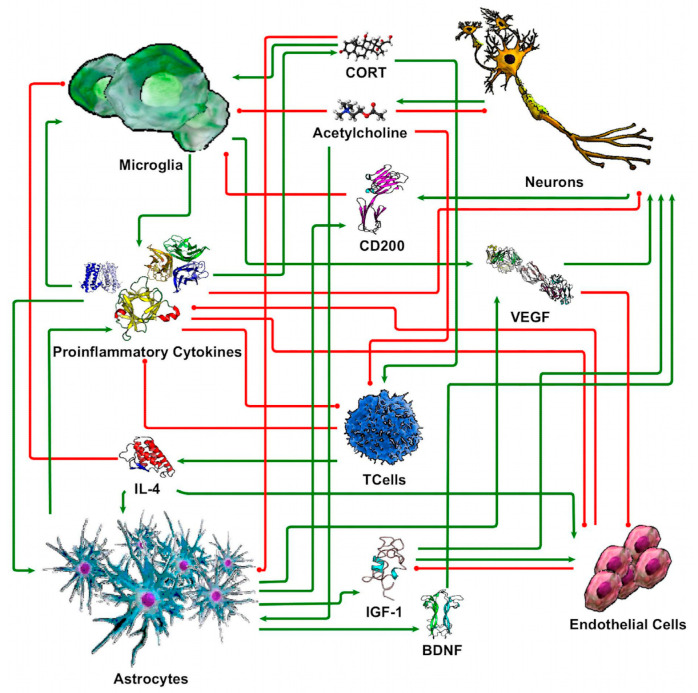
Simple neuroimmune signaling network. Connections with green arrow terminators represent stimulatory effects, while connections with red circle terminators represent inhibitory effects. From Craddock et al. 2018 [33].

Following a previously constructed discrete ternary logic model of neuronal-glial interaction [33], the elements of a neuroimmune system model (Figure 2) were represented in terms of 3 discrete states: −1 (below typical health), 0 (typical health), and 1 (above typical health). The number and qualitative signature of stable regulatory patterns available to a model system of neuron–glia interaction (Figure 2) were determined by applying a discrete ternary formalism [38] to the system. In brief, the evolution of each state of the system in time was assessed such that each element in the system will increase at the next point in time when there are more stimulating signals and decrease when there are only inhibiting signals at the current time point. The situation where the logic indicates that the current state prefers to evolve toward itself indicates that the current state is a stable state of the system (i.e., it does not evolve in time). The qPCR gene expression neuroimmune profiles from the TBI rat model were then compared to each model-predicted steady-state behavior of the neuroimmune system through the application of Brown’s theoretical approximation [39] of Fisher’s statistics, as conducted in our previous work [33,38,40,41,42,43]. This method was chosen as it provides a meta-analysis technique to combine non-independent probabilities and obtain an overall significance measure P based on a set of *p*-values obtained from independent *t*-tests. The aggregate value P ranges between 0 and 1, with 0 indicating complete overlap and 1 being the farthest distance from a stable state. This method is applicable as the model elements do not express independently, as evidenced by the connectivity of the neuron–glia interaction model (Figure 2). The above-mentioned qPCR data were compared against the model predicted states based on the 7 measured variables: IL-1β, IL-6, and TNFα as representing the proinflammatory cytokine node, GFAP denoting astrocyte status, and CCL2, LIF, and OSM denoting microglia activation. Where model variables represent an aggregate set of markers, each experimentally measured constituent marker was compared individually to the model-predicted value. Sammon’s nonlinear mapping criterion [44] was used to project the *p*-value distances onto a 2-dimensional plot and describe the statistical significance of separation between measured and predicted co-expression patterns. 

### 2.8. Statistics

Sample size was determined to be at least an *N* = 4/group based on previous calculations related to the measurement of cytokine mRNA in brain tissue [36]. An *N* = 5/group was used for this study to account for potential TBI-related mortality and statistical or technical outliers, e.g., poor quality of formalin perfusion. Statistical outliers were identified using Grubb’s test (α = 0.05) (GraphPad QuickCalcs: https://www.graphpad.com/quickcalcs/Grubbs1.cfm; accessed on 8 November 2022). A 1-way ANOVA analysis with Fisher’s LSD post hoc test was performed using SigmaPlot (v. 14.0; Systat Software, Inc., San Jose, CA, USA) with statistical significance set at *p* ≤ 0.05. Data from qPCR were log-transformed prior to one-way ANOVA, as they did not follow a normal distribution.

## 3. Results

### 3.1. Impact with a Stainless-Steel Projectile Induced Significantly Prolonged Periods of Unconsciousness and Neurobehavioral Impairment Compared to an Aluminum Projectile

Previous studies utilizing the WRAIR PCI model of TBI have employed a helmet to produce diffused impacts and prevent the severe trauma observed in the initial studies [23,31]. However, not all TBIs occur when protective equipment is used. Therefore, the aim was to develop a modified PCI model that would successfully produce a non-penetrating TBI in the absence of a helmet to serve as a baseline for future studies. Here, we employed a stainless-steel ball-bearing projectile similar to that described in the WRAIR PCI model [23], as well as an aluminum ball bearing that had significantly less mass than the stainless-steel ball bearing. As such, we found that the stainless-steel projectile produced more prominent clinical signs of sTBI in the rats, including an increased risk for hematoma (5/8), skull fracture (3/8), acute porphyrin staining (2/8), and mortality (2/10) (Table 1). However, the rats receiving an impact with the aluminum ball-bearing projectile appeared largely similar to sham rats, with only 1 rat displaying acute porphyrin staining within the first 24 h following TBI (Table 1). 

**Figure 3:**
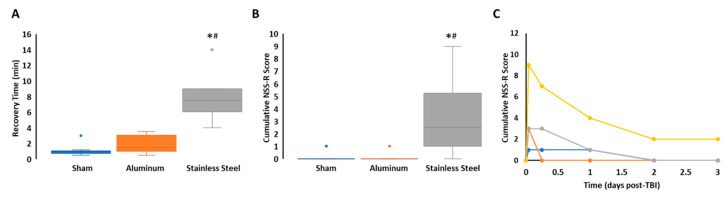
Impact with the stainless-steel ball bearing significantly impacted recovery time and neurobehavior following TBI. Rats were subjected to TBI using the projectile concussive impact model with either an aluminum or stainless-steel ball bearing or sham (no ball bearing). (**A**) Recovery times were recorded for each rat from the time of removal from the anesthesia induction chamber to regaining consciousness following impact. (**B**) Rats were subjected to neurobehavioral testing at 1 h following TBI, and cumulative scores were calculated. (**C**) Timeline of cumulative neurobehavioral scores at baseline, 1 h, and 1–3 days post-TBI for rats receiving TBI with the stainless-steel ball bearing. *p* ≤ 0.05 vs. * Sham or # Aluminum.

In addition to these gross observations, rats that were impacted with the stainless-steel projectile had a significantly longer recovery time from anesthesia compared to the aluminum projectile and sham rats (Figure 3A). While not statistically significant, rats receiving a TBI from the aluminum projectile remained unconscious for an average of 43 s longer than the shams. Rats that received a TBI with the stainless-steel projectile also had a significantly higher score on the NSS-R at 1 h post-TBI compared to those exposed to the aluminum projectile or sham conditions (Figure 3B). At this time point, the stainless-steel-impacted rats displayed deficits in general balance, eye reflex, landing, and sound reflex, as well as displaying grasp deficiencies. Of the 4 surviving rats in the stainless-steel group observed until 72 h post-TBI, all but 1 rat recovered to baseline neurobehavioral scores by 48 h post-TBI (Figure 3C).

### 3.2. Impact with a Stainless-Steel Projectile Produced Significant Neuronal Damage and Gliosis Compared to an Aluminum Projectile

**Figure 4:**
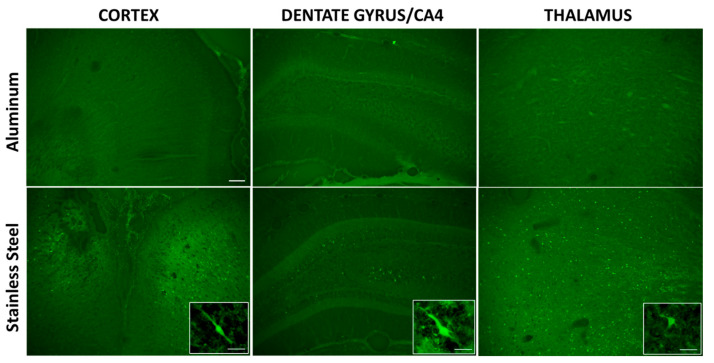
TBI induced by impact with the stainless-steel ball bearing produced significant neuronal damage. Rats were subjected to TBI using the projectile concussive impact model with either an aluminum or stainless-steel ball bearing or sham (no ball bearing; not shown). Neuronal damage was evaluated at 72 h post-TBI in histological brain sections using FluroJadeB (FJB). FJB+ cells were observed in the cortex, dentate gyrus/CA4 regions of the hippocampus, and the thalamus. Representative images captured at 10× magnification (scale bars representative of 100 μm), and inset images were captured at 60× magnification (scale bars representative of 20 μm).

TBI is known to be associated with neuronal damage, neurodegeneration, and neuroinflammation. To measure these responses, we examined neuronal damage and gliosis histologically. Using Fluoro-Jade B (FJB) stain to visualize degenerating neurons, it was found that rats impacted with the stainless-steel projectile had FJB+ cells in the cortex, the hippocampus (dentate gyrus, CA4, and CA3 regions), and throughout the thalamus, while rats impacted with the aluminum projectile showed no FJB+ staining (Figure 4); sham rats also had no FJB+ staining. For rats impacted with the stainless-steel projectile, the highest density of FJB+ cells found in the cortex was along the posterior midline, correlating with the site of impact.

**Figure 5:**
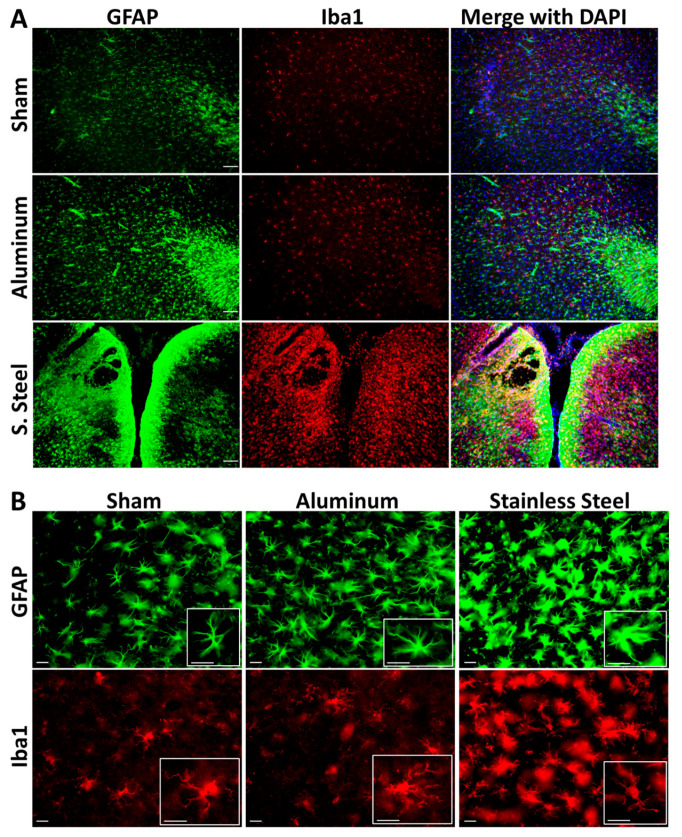
TBI produced astrogliosis and microgliosis with a more significant effect from impact with the stainless-steel ball bearing. Rats were subjected to TBI using the projectile concussive impact model with either an aluminum or stainless-steel ball bearing or sham (no ball bearing). Gliosis was evaluated in the cortex at 72 h post-TBI in histological brain sections using GFAP (astrocytes) and Iba1 (microglia). Representative images captured at 10× ((**A**); scale bars representative of 100 μm) or 40× ((**B**); scale bars representative of 20 μm) magnification.

Co-immunostaining with the astrocyte and microglial markers GFAP and Iba1, respectively, showed that the same brain areas that contained FJB+ cells in the stainless-steel-impacted rats also displayed extensive astrocyte and microglial reactivity. This included the posterior midline region of the cortex (Figure 5A) as well as the hippocampus and thalamus (Supplemental Materials Figure S1). Interestingly, while the rats that received TBI with the aluminum projectile did not display neuronal damage, this condition did produce mild astrocyte and microglial reactivity. While these rats had minor subjective increases in the intensity of GFAP and Iba1 staining (Figure 5A), closer inspection of the astrocytes and microglia from these rats indicated that they were activated in comparison to Shams but did not display as significant structural changes as those observed in rats that received TBI with the stainless-steel projectile (Figure 5B). The activated astrocytes in the stainless-steel group display significant hypertrophy of the cells and a loss of the finer astrocytic processes, while those in the aluminum group show slightly less hypertrophy and retain some finer processes (Figure 5B). The microglia of the stainless-steel group are hypertrophied and display thicker and shorter processes compared to shams, while the microglia of the aluminum group show hypertrophied cell bodies with slightly longer, filamentous processes compared to those of the stainless-steel group (Figure 5B). These observations suggest that while only the stainless-steel projectile produced neuronal damage, both projectiles were capable of producing neuroinflammatory gliosis. 

### 3.3. Impact with a Stainless-Steel Projectile Induced Significant Increases in the Expression of Inflammatory Cytokine mRNA in Several Brain Areas Compared to an Aluminum Projectile

**Figure 6:**
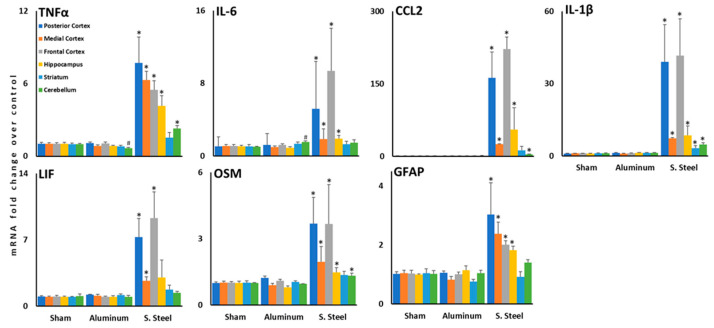
Impact with the stainless-steel ball bearing significantly increased the expression of inflammatory molecules in the brain. Rats were subjected to TBI using the projectile concussive impact model with either an aluminum or stainless-steel ball bearing or sham (no ball bearing). The inflammatory cytokines and chemokines TNFα, IL-6, CCL2, IL-1β, LIF, OSM, and the astrocyte marker GFAP were measured in the cortex (separated into posterior, medial, and frontal sections), hippocampus, striatum, and cerebellum. *p* ≤ 0.05 vs. * Sham and Aluminum or # Sham. Data shown for cortical areas combine values from left and right hemispheres.

As it was found that TBI instigated by both projectiles produced gliosis and it is known that microglia and astrocytes are sources of inflammatory cytokines and chemokines in the brain, regional levels of cytokine and chemokine mRNA were measured in the brain. As expected, the rats in the stainless-steel group, which displayed neuronal degeneration and significant gliosis (Figure 4 and Figure 5), also had significant increases in the expression of cytokine, chemokine, and GFAP mRNAs (Figure 6). Specifically, TNFα, IL-6, CCL2, IL-1β, LIF, OSM, and GFAP mRNAs were increased over both the aluminum-impacted and sham rats in all areas of the cortex, and all mRNAs, except for LIF, were significantly increased in the hippocampus; all areas of the brain that demonstrated neurodegeneration and significant gliosis were in the stainless-steel group. While several cytokines and chemokines showed increased mRNA expression in the cerebellum (TNFα, CCL2, IL-1β, and OSM), only IL-1β mRNA was found to be increased in the striatum, which is anatomically distant from the site of impact (Figure 6). Interestingly, while rats in the group receiving TBI with the aluminum ball bearing displayed morphological changes to both astrocytes and microglia, the expression of inflammatory mediators was largely unaltered by this exposure, aside from a slight increase in the expression of IL-6 mRNA in the cerebellum (Figure 6). Taken together, these observations suggest that impact with the stainless-steel ball bearing produces an sTBI, while the aluminum ball bearing generates an mTBI.

### 3.4. sTBI Closely Aligns with the Neuroinflammatory State in a Computational Neuron-Glia Interaction Network

**Figure 7:**
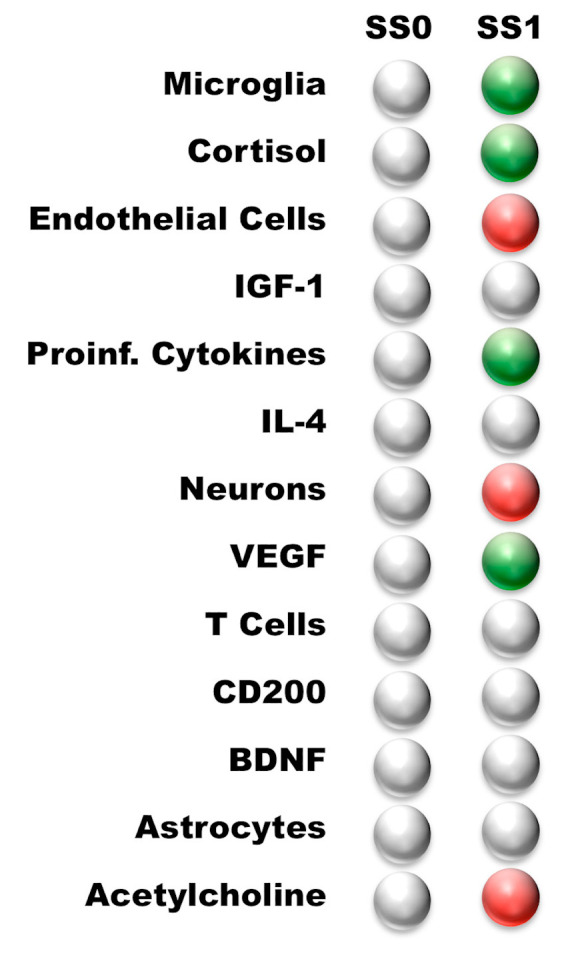
Steady states predicted by the neuro-immune model. White—nominal state (0); Green—high state (1); Red—low state (−1). The steady states are named according to their profile: Typical Health (SS0) and Neuroinflammation (SS1). From Craddock et al. 2018 [33].

Previous application of discrete ternary logic assessment of a neuron-glia interaction model (Figure 2) predicted two stable regulatory patterns within the neuroimmune system (Figure 7), as previously reported [33]. The first was indicative of typical health with all system elements at nominal health values (SS0). The second was indicative of a neuroinflammatory state characterized by increased activation of microglia, elevated levels of cortisol, proinflammatory cytokines, and vascular endothelial growth factor (VEGF), decreased health of neurons, a weakened blood–brain barrier, and decreased levels of acetylcholine (SS1).

**Figure 8:**
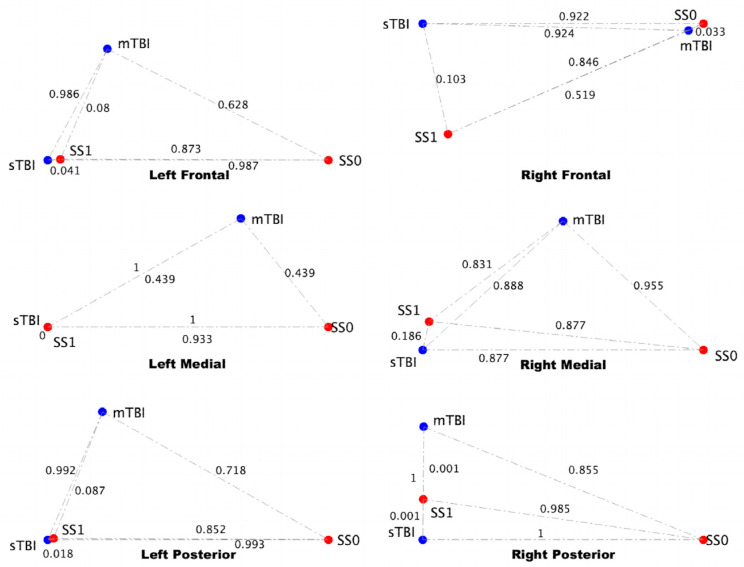
Alignment of brain injury conditions compared to sham controls with model-predicted stable states. Sammon projection in two dimensions for gene expression obtained in frontal, medial, and posterior cortex in the left and right brain hemispheres. Red dots represent the model-predicted stable states (SS0 and SS1). Blue dots represent the aggregated gene expression data for mTBI (aluminum ball bearing), and sTBI (stainless-steel ball bearing) vs. sham controls. Axes represent arbitrary units such that the relative distance between points approximates the aggregated *p*-values between all points.

Evaluating how the neuroinflammatory profiles produced by the two TBI conditions used in this study align with the steady states of the neuron–glia interaction model, the mRNA expression data for the cortical regions (Figure 6) was projected onto the model. From the alignment (Figure 8), it can be seen that in all regions, the sTBI condition most closely aligns with the neuroinflammatory state (SS1) and is positioned far (close to 1) away from the typically healthy state (SS0). The mTBI condition most closely aligns with the neuroinflammatory state (SS1) in the posterior region, is roughly equidistant from health and neuroinflammation in the medial region, and aligns with typical health in the right frontal cortex region but with the neuroinflammatory state in the left frontal cortex region. In all cases, the mTBI state is different from the sTBI state owing to the large differences in their expression levels. 

## 4. Discussion

In this study, we have found that focal TBI produced using the PCI model of closed-head injury in the absence of a protective helmet is capable of producing neuroinflammation and/or neuronal damage across various regions of the brain. The severity of TBI produced using this model can be varied based on the material/mass of the ball-bearing projectile; here, we found that an aluminum projectile produced mTBI, while a stainless-steel projectile produced sTBI. The sTBI condition was characterized by an increased risk for mortality, prolonged unconsciousness, significant neurocognitive impairment, widespread neuronal damage, and robust neuroinflammatory responses. In contrast, the mTBI condition resulted in minimal periods of unconsciousness, no neurocognitive impairment or neuronal damage, and mild activation of microglia and astrocytes; however, despite the mild nature of the response, glial activation was observed across different areas of the brain, including the cortex, hippocampus, and thalamus. Moreover, the alignment of the two TBI models with our previously defined neuron–glia interaction computational model indicated that sTBI was more closely associated with a pathological neuroinflammatory state, while the mTBI condition fell between the neuroinflammatory and health steady states.

Head injuries can result in TBIs of varying severity, and, clinically, these present with different acute symptoms. sTBI results in significant impairment with prolonged periods of unconsciousness and increased risk of death, as well as cognitive, memory, sensory, motor, and mood disturbances. In our model, impact with the stainless-steel projectile directly to the head produced immediate symptoms that closely aligned with the symptoms of sTBI in humans (Table 1 and Figure 3). In contrast, the aluminum projectile produced significantly milder symptoms that were largely indiscernible from the sham-treated controls (Table 1 and Figure 3). These milder symptoms, or lack-there-of, align well with the symptoms of mTBI in humans, with the rats showing greater variability in unconsciousness following mTBI compared to Sham (Figure 3A) in the absence of neurobehavioral impairment (Figure 3B). This lack of significant post-TBI symptoms aligns with speculation that many cases of mTBI in humans go unnoticed and undiagnosed [3,8]. 

In our models of mTBI and sTBI, we saw varying levels of neuroinflammation and damage. In particular, sTBI produced by the stainless-steel projectile resulted in a significant elevation of inflammatory cytokine mRNAs across several areas of the brain, including the cortex, hippocampus, and cerebellum within 6 h of injury (Figure 6), which was accompanied by significant gliosis, involving both astrocytes and microglia and neuronal cell death observed 3 days following TBI (Figure 4 and Figure 5). These findings correlate with the significant primary damage to the brain that can occur in sTBI, which is more likely to result in the death of neurons. However, our results also indicate that the inflammatory mechanisms associated with this damage can happen within a matter of hours following injury, in addition to a more protracted change in the inflammatory state of the brain over a period of days. Interestingly, mTBI in our model did not produce any measurable changes in cytokine mRNA acutely following injury but produced observable changes in astrocytes and microglia days later in the absence of neuronal damage. These glial changes could be the result of a delayed response to the less severe axonal damage or shearing that can result from mTBI [8], which can lead to axonal degeneration and long-term neurological dysfunction [45,46], or as a response to primary injury of glia [12]. It is possible that a later time point would need to be evaluated to measure changes in cytokine mRNA in our mTBI model. Moreover, despite the potential for axonal damage in mTBI, there was no indication of neuronal degeneration (Figure 4). While FluoroJade B is capable of staining the axons of degenerating neurons [47], it is unclear whether this degenerative stain is capable of staining damaged axons in the absence of the entire neuron degenerating. As such, potential FluoroJade B staining of axons without staining of the soma was observed in the dentate gyrus following mTBI in our model, however, similar staining was also observed in Sham-treated rats (Supplemental Materials Figure S2). Further investigation using a more specific stain for axonal degeneration, such as silver staining [48], may better identify axonal damage and degeneration in our mTBI model. Lastly, our findings that the cortex, hippocampus, thalamus, and cerebellum were the brain areas most affected by TBI are in line with previous studies that have also found involvement of these brain areas [23,24,25,28,49,50]. 

Previously, we established a computational model describing neuron-glia interactions associated with healthy and chronic neuroinflammatory states [33]. In the current study, the cytokine mRNA profiles from mTBI and sTBI were applied to this model and evaluated for their alignment to either the aberrant neuroinflammatory state or healthy neuroimmune function (Figure 8). Unsurprisingly, due to the significantly exacerbated neuroinflammatory response following sTBI, this condition is strongly associated with the neuroinflammatory state across all areas of the cortex, an observation that correlates with the severe outcomes and extensive neuroinflammatory response associated with more severe incidences of TBI. Interestingly, the mTBI condition largely fell between the neuroinflammatory and healthy states, except in the right posterior region of the cortex which aligned more closely to the neuroinflammatory state and was closer to the site of impact of the projectile. This suggests that while the brain is not “healthy” following an mTBI, it fails to fully precipitate a robust neuroinflammatory response. This mild neuroinflammatory state following a single mTBI could make the brain more vulnerable to future insult(s), which could explain the increased risk for more significant post-injury outcomes in individuals experiencing multiple mTBIs [51,52]. 

## 5. Conclusions

Overall, we have demonstrated that mTBI and sTBI can be experimentally modeled using the PCI animal model of TBI in the absence of a helmet by varying the material of the projectile, e.g., aluminum versus stainless steel, respectively. Not only do these models accurately reproduce the clinical and experimental manifestations associated with TBI of neurobehavioral dysfunction, neuroinflammation, and neural damage, but they also align with the computational neuron–glia interaction model of neuroinflammation on a spectrum relative to the severity of the injury. These highly reliable and reproducible TBI models have significant potential for the continued evaluation of the behavioral, cellular, and molecular outcomes associated with TBI, as well as measuring the efficacy of novel helmet materials in preventing brain injury. 

## Figures and Tables

**Table 1:** Post-TBI Clinical Observations.

Group	Total *N*	Mortality ^1^	Acute Porphyrin ^2^	Hematoma	Fracture
Sham	10	0	0	0	0
Aluminum	10	0	1	0	0
Stainless Steel	10	2	2	5	3

^1^ Rats never regained consciousness from anesthesia. ^2^ Only observed within the first 24 h post-TBI.

## Data Availability

All raw data is available on the NIOSH Data and Statistics Gateway (https://www.cdc.gov/niosh/data/).

## Supplementary Materials

The following supporting information can be downloaded at: https://www.mdpi.com/article/10.3390/brainsci13040623/s1, Figure S1: TBI produced astrogliosis and microgliosis in the hippocampus and thalamus; Figure S2: FluoroJade B staining of axons in the dentate gyrus of Sham and mTBI rats.
