# Supplementary material for: A Projectile Concussive Impact Model Produces Neuroinflammation in Both Mild and Moderate-Severe Traumatic Brain Injury
Source: Brain Sci. 2023 Apr 6;13(4):623. doi: 10.3390/brainsci13040623 (PMC10136957; doi:10.3390/brainsci13040623)
Supplement: Supplementary file 1 [file brainsci-13-00623-s001.zip › Supplemental Figure Legends_rev.pdf]

A Projectile Concussive Impact Model Produces Neuroinflammation in Both Mild and Moderate-Severe Traumatic Brain Injury

Lindsay T. Michalovicz [1,*], Kimberly A. Kelly[1], Travis J. A. Craddock[2,3,4,5] and James P. O'Callaghan[1]

[1]Centers for Disease Control and Prevention, Health Effects Laboratory Division, National Institute for Occupational Safety and Health, Centers for Disease Control and Prevention, Morgantown, WV, 26508, USA;

[2]Institute for Neuro-Immune Medicine, Nova Southeastern University, Fort Lauderdale , FL, 33314, USA;

[3]Department of Clinical Immunology, College of Osteopathic Medicine, Nova Southeastern University, Fort Lauderdale, FL, 33314, USA

[4]Department of Psychology & Neuroscience, College of Psychology, Nova Southeastern University, Fort Lauderdale, FL, 33314, USA

[5]Department of Computer Science, College of Engineering and Computing, Nova Southeastern University, Fort Lauderdale, FL, 33314, USA

[*]Correspondence: yqp4@cdc.gov

Supplemental Figure Legends

Supplemental Figure S1. TBI produced astrogliosis and microgliosis in the hippocampus and thalamus. Rats were subjected to TBI using the projectile concussive impact model with either an aluminum or stainless steel ball bearing, or sham (no ball bearing). Gliosis was evaluated in the hippocampus (A) or thalamus (B) at 72 hours post-TBI in histological brain sections using GFAP (astrocytes) and Iba1 (microglia). Representative images captured at 10x magnification (scale bars representative of 100 µm).

Supplemental Figure S2. FluoroJade B staining of axons in the dentate gyrus of Sham and mTBI rats. Rats were subjected to TBI using the projectile concussive impact model with an aluminum ball bearing or sham (no ball bearing). Axonal staining was visualized at 72 hours post-TBI in histological brain sections using FluoroJade B. Representative images captured at 60x magnification (scale bars representative of 20 µm).
