# Supplementary figures and images for: A Projectile Concussive Impact Model Produces Neuroinflammation in Both Mild and Moderate-Severe Traumatic Brain Injury
Source: Brain Sci. 2023 Apr 6;13(4):623. doi: 10.3390/brainsci13040623 (PMC10136957; doi:10.3390/brainsci13040623)

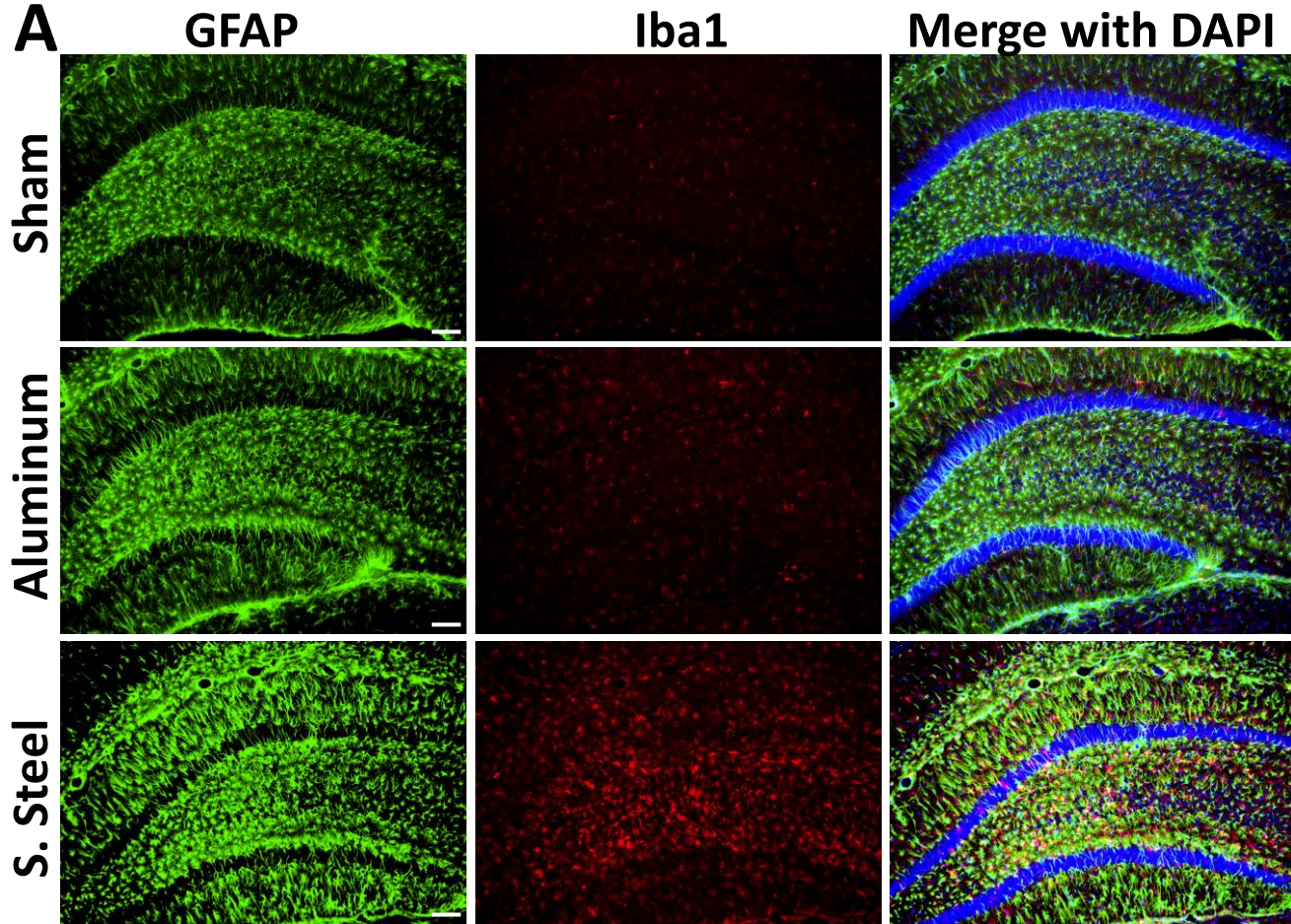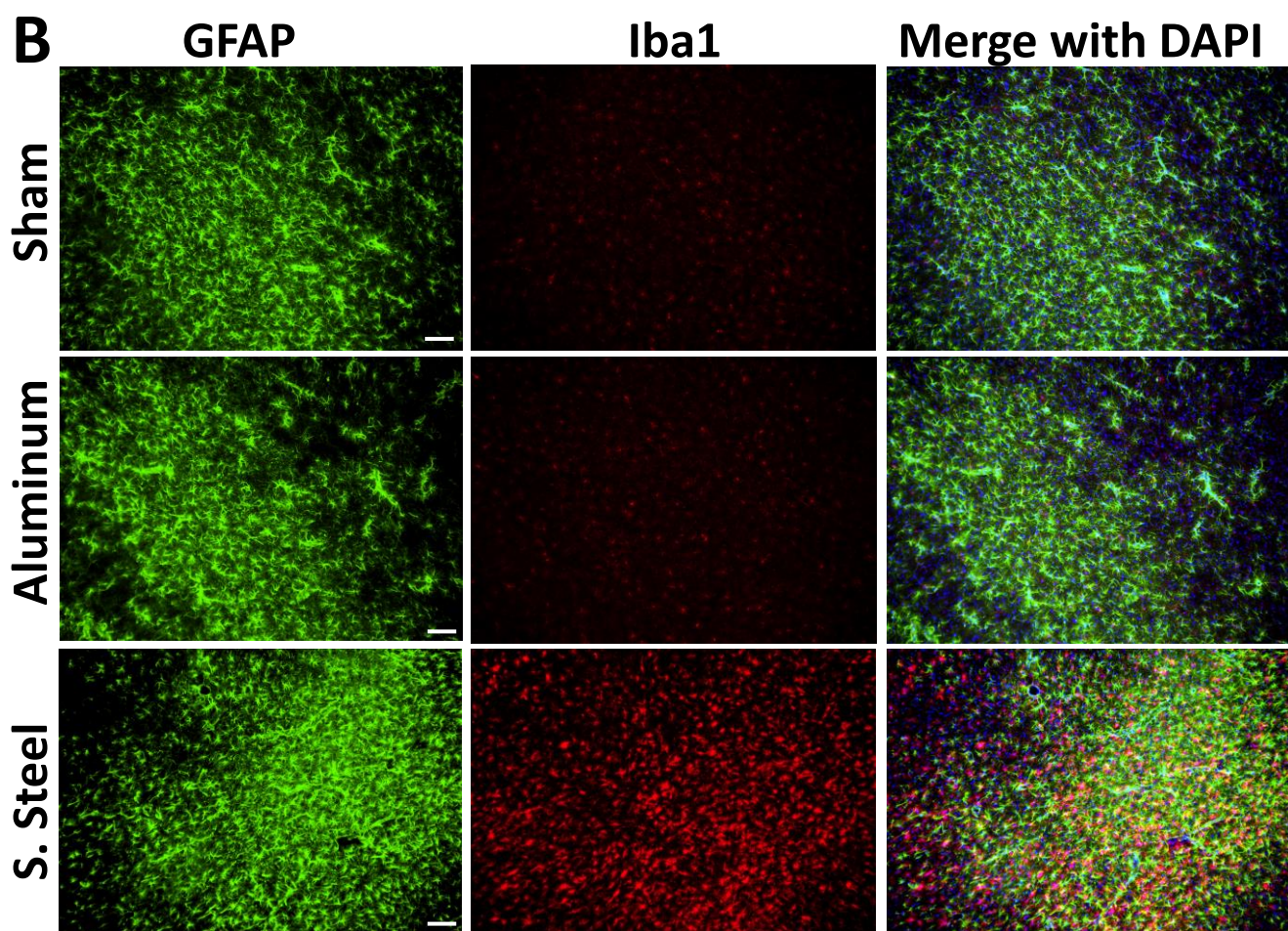

Supplement: Supplementary file 1 [file brainsci-13-00623-s001.zip › Supp Figure 1.pdf]

**Sham**

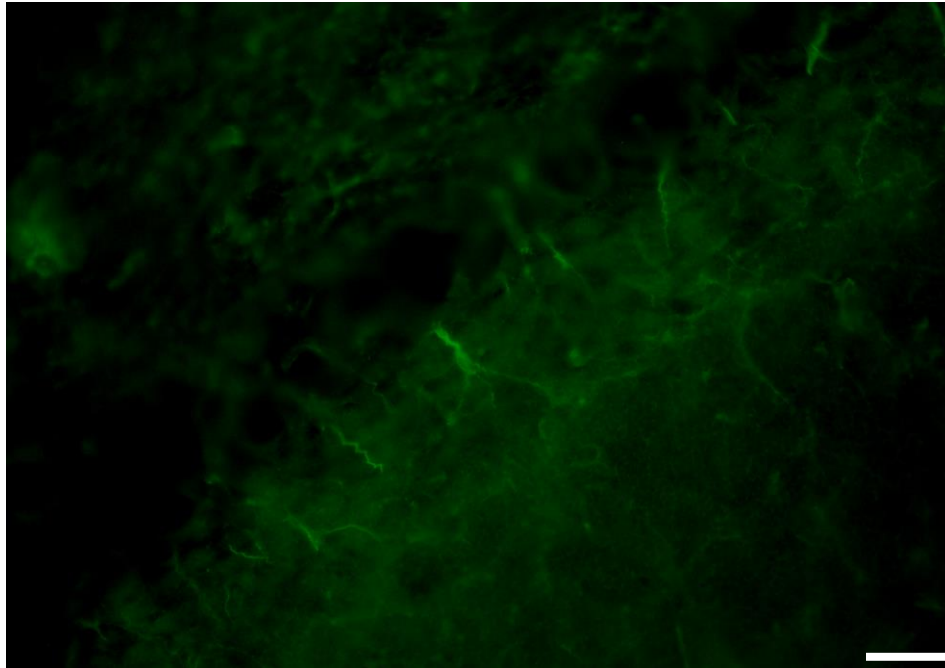

**Aluminum**

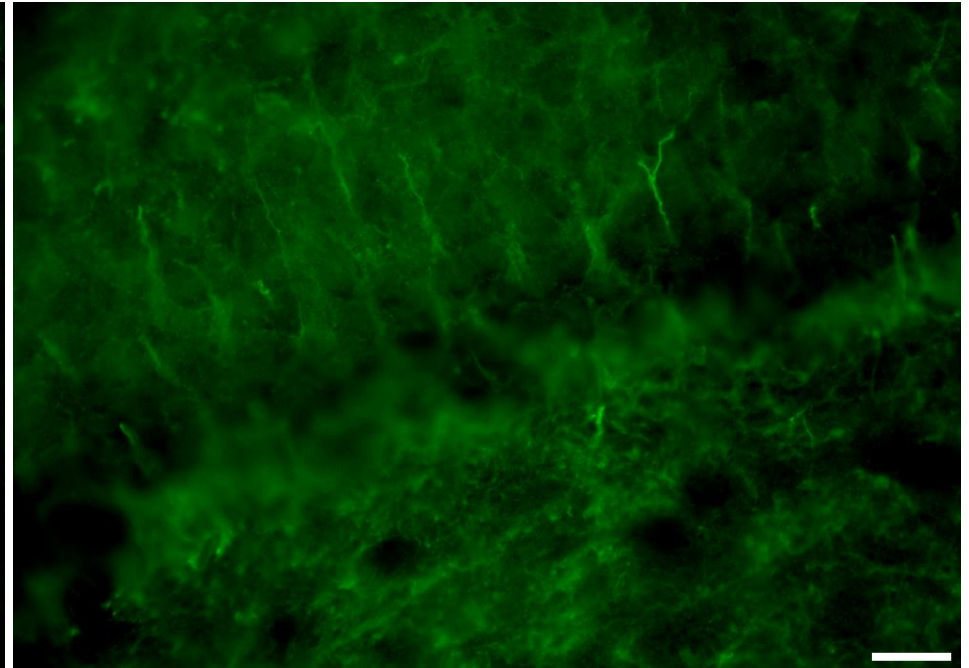

Supplement: Supplementary file 1 [file brainsci-13-00623-s001.zip › Supp Figure 2.pdf]
